# Role of Pneumococcal NanA Neuraminidase Activity in Peripheral Blood

**DOI:** 10.3389/fcimb.2019.00218

**Published:** 2019-06-26

**Authors:** Shahan Syed, Pipsa Hakala, Anirudh K. Singh, Helena A. K. Lapatto, Samantha J. King, Seppo Meri, T. Sakari Jokiranta, Karita Haapasalo

**Affiliations:** ^1^Department of Bacteriology and Immunology, University of Helsinki, Helsinki, Finland; ^2^Center for Microbial Pathogenesis, The Abigail Wexner Research Institute at Nationwide Children's Hospital, Columbus, OH, United States; ^3^Department of Microbiology, Medical College, All India Institute of Medical Sciences, Bhopal, India; ^4^Department of Pediatrics, The Ohio State University, Columbus, OH, United States; ^5^SYNLAB Finland, Helsinki, Finland

**Keywords:** pneumococcus, sialidase, alternative pathway, pneumonia, inflammation

## Abstract

The most frequent form of hemolytic-uremic syndrome (HUS) is associated with infections caused by Shiga-like toxin-producing Enterohaemorrhagic *Escherichia coli* (STEC). In rarer cases HUS can be triggered by *Streptococcus pneumoniae*. While production of Shiga-like toxins explains STEC-HUS, the mechanisms of pneumococcal HUS are less well-known*. S. pneumoniae* produces neuraminidases with activity against cell surface sialic acids that are critical for factor H-mediated complement regulation on cells and platelets. The aim of this study was to find out whether *S. pneumoniae* neuraminidase NanA could trigger complement activation and hemolysis in whole blood. We studied clinical *S. pneumoniae* isolates and two laboratory strains, a wild-type strain expressing NanA, and a NanA deletion mutant for their ability to remove sialic acids from various human cells and platelets. Red blood cell lysis and activation of complement was measured *ex vivo* by incubating whole blood with bacterial culture supernatants. We show here that NanA expressing *S. pneumoniae* strains and isolates are able to remove sialic acids from cells, and platelets. Removal of sialic acids by NanA increased complement activity in whole blood, while absence of NanA blocked complement triggering and hemolytic activity indicating that removal of sialic acids by NanA could potentially trigger pHUS.

## Introduction

*Streptococcus pneumoniae* infection is a major cause of morbidity and mortality worldwide. Despite the current vaccination program it kills approximately half a million children under 5 years of age worldwide every year. It typically causes diseases such as otitis media, pneumonia, bacteremia, and meningitis. Pneumococcal atypical hemolytic uremic syndrome (pHUS) is a rare complication of an invasive pneumococcal infection that mainly affects young children (Waters et al., 2007). *S. pneumoniae* is known to express neuraminidases, NanA, NanB, and NanC that can remove sialic acids from cell surfaces (Burnaugh et al., 2008). Of these, *nanA* and *nanB* genes are present in almost all clinical *S. pneumoniae* isolates while *nanC* is present in ~50% of isolates (Pettigrew et al., 2006). Removal of sialic acids from cell membrane glycostructures also reduces binding of complement regulator factor H to self-cell surfaces (Nissila et al., 2018). This may lead to a defect in complement regulation on autologous cells similarly as in a rare form of HUS called atypical HUS (aHUS) (Szilagyi et al., 2013; Jokiranta, 2017).

The complement system is composed of more than 40 soluble and cell surface anchored proteins (Sarma Ward and Ward, 2011). It targets microbes and damaged self-cells via recognition of foreign or exposed self-antigens by antibodies (classical) or carbohydrate recognizing lectins (lectin pathway), or by spontaneous hydrolysis of C3 (alternative pathway, AP) (Jokiranta, 2017). All three pathways lead to opsonization of the target surface by C3b, generation of chemotactic fragments, C3a and C5a, and formation of membrane attack complexes (MAC, C5b-9) that can directly lyse the target (Sarma Ward and Ward, 2011). Because C3b can deposit on any biological surface, it can also deposit on the host's own cells. Therefore, strict regulation of its activation is essential.

Factor H is a key regulator of the AP. It is composed of 20 domains from which domains 5–7 bind C-reactive protein, apolipoprotein E, and negatively charged polyanions like heparin 7 (Blackmore et al., 1996; Giannakis et al., 2003; Haapasalo et al., 2015) while domains 1–4 and 19–20 bind to C3b. The C-terminal domains 19–20 mediate simultaneous binding to deposited C3b and cell surface sialic acids (Kajander et al., 2011). Factor H recognizes α2–3 linked N-terminal sialic acid glycans that are found abundantly on various human cells (Blaum et al., 2015). These interactions explain factor H-mediated discrimination between self and non-self cells. The importance of factor H-mediated self-surface recognition is exemplified by the development of aHUS when mutations in factor H or anti-factor H autoantibodies disturb the domain 19–20 mediated interaction with sialic acids and/or C3b (Hyvarinen et al., 2016).

The aHUS-associated mutations in domains 19–20 of factor H significantly reduce the interaction between factor H and sialic acids on red blood cells, endothelial cells, and platelets (Hyvarinen et al., 2016). This partially explains the molecular mechanism behind the severe endothelial cell damage caused by FH mutations in aHUS. Most aHUS cases can be explained by mutations in complement genes or autoantibodies against factor H. However, ~40% of aHUS cases do not have an explanation (Noris et al., 2014). Many of these secondary HUS cases are caused by infections with microbes other than Shiga-like toxin-producing Enterohemorrhagic *E. coli* (STEC) such as influenza virus and *S. pneumoniae* (Szilagyi et al., 2013;Bitzan and Zieg, 2017).

The present study was set up to investigate, whether removal of cell surface sialic acids by *S. pneumoniae* NanA could play a role in triggering pHUS. We show here that the presence of NanA in *S. pneumoniae* culture supernatant removes sialic acids from various cell types. The release of sialic acid residues increases hemolysis and complement activation in whole blood and activates platelets as well. The presence of NanA in whole blood and in the presence complete microbial secretome suggests a significant role for NanA in uncontrolled complement-mediated hemolysis and platelet aggregation.

## Materials and Methods

### Bacterial Strains and Growth Conditions

Preparation of S*. pneumoniae* strains D39 wt and D39 Δ*nanA* (serotype 2) have been described (King et al., 2004). Isolates 1, 2, and 3 were S*. pneumoniae* serotype two strains isolated from a blood culture of septic patients with the permission of the ethical review board of the Hospital District of Helsinki and Uusimaa, Finland (448/13/03/00/09). Bacteria were grown in Todd Hewitt Broth (THB) in 5% CO_2_ at 37°C until late log-phase (OD620 ~0.7) and centrifuged at 3,000 × g for 10 min. Supernatants were filtered through 0.45 μm filters (Nalgene, Rochester, NY, US) and stored at −80°C.

### Proteins

Neuraminidase from *Clostridium perfringens* (defined as Neu in the manuscript) was purchased from New England Biolabs (Ipswich, MA, US). The recombinant NanA neuraminidase (defined as RecNanA in the manuscript) containing only the catalytic region (aa303-777) of *S. pneumoniae* NanA (SP_1693) was expressed with N-terminal 6xHis tag. The DNA was amplified using forward *AAGTTCTGTTTCAGGGCCCGCCTGAAGGAGCGGCTTTAAC* and reverse *ATGGTCTAGAAAGCTTTAATTTTTGCTCAAAAATTCCCA* primers and cloned in pOPINF vector. For expression plasmids were transformed in *E. coli* Rosetta DE3 cells. Four hundred milliliter of LB with 100 μg/ml ampicillin was inoculated with 8 ml of overnight culture grown in LB ampicillin. Cells were grown to OD600 ~0.8 at 37°C with shaking and afterward culture was transferred to 25°C and grew overnight. Cells were harvested and resuspended in 20 ml of binding buffer (20 mM Sodium phosphate buffer, pH 7.4, 5 mM imidazole, 500 mM NaCl, 5 mM β-mercaptoethanol) and lysed using French press. Supernatant was collected by centrifugation at 30,000 × g for 30 min at 4°C. Protein was allowed to bind to ProBond Ni-NTA agarose (Invitrogen) for 45 min at 4°C. Column was washed with binding buffer containing 20 mM imidazole and eluted in 8 ml of elution buffer (binding buffer with 300 mM imidazole).

### Cell Culture

Human embryonic kidney cells (HEK293T) were cultured in Dulbecco's Modified Eagle's Medium (DMEM) supplemented with 10% fetal bovine serum, penicillin, streptomycin, and L-glutamine (Gibco, Waltham, MA, US) as described (Haapasalo et al., 2018). Human umbilical endothelial cells (HUVECs) were cultured in EndoGRO-LS media (Millipore, Burlington, MA, US) as described (Hyvarinen et al., 2016) and passaged ≤6 in our assays. ATCC U937 human monocytic cells were obtained as a kind gift from Dr. Carla de Haas (UMCU, Netherlands) and cultured in Roswell Park Memorial Institute (RPMI) 1640 medium (Gibco, Waltham, MA, US) supplemented with penicillin, streptomycin and 10% (v/v) fetal bovine serum.

### Neuraminidase Activity and Sialic Acid Removal Assay

2′-(4-Methylumbelliferyl)-α-D-N-acetylneuraminic acid (MUAN) sodium salt hydrate (Sigma, St. Louis, MI, US) was used to measure the activities of RecNanA (at 10 nM concentration), Neu (at 110 nM final concentration), and supernatants (10 μl) from *S. pneumoniae* strains D39 wt and D39 ΔnanA and the clinical isolates. Briefly a 0.2% solution of MUAN (2′-(4 –methyl-umbelliferyl) α-D-N-acetylneuraminic acid) was made in 0.25 M Sodium acetate buffer pH 7. Pure proteins or culture supernatants were mixed with 10 μl of MUAN and incubated at 37°C for 5 min. 1.5 ml of sodium carbonate buffer pH 9.6 was added and mixed. Two hundred microliter of the mixture was placed in a black well-microtitre plate (NUNC) for 1 s reading at 355 nm/460 nm with a 5 s shake of the plate. Negative control used was reaction using media but no bacterial cells or 150 mM phosphate buffered saline (pH 7.4) (PBS).

A modified thiobarbituric acid assay was used to analyze the released sialic acids from cell surfaces as previously described (Warren, 1959). Mammalian cells were incubated with 10% (v/v) bacterial supernatant for 30 min at 37°C in RPMI supplemented with 0.05% human serum albumin (RPMI-HSA) (Sigma). The pelleted supernatant (300 × g for 10 min) from this reaction was oxidized by addition of 1 ml sodium arsenate (10% wt/vol) in 0.5 M sodium sulfate/0.05 M H_2_SO_4_ (Sigma). The chromophore was developed by addition of 3 ml of 2-thiobarbituric acid (0.6% wt/v) in 0.5 M sodium sulfate (Sigma). Samples were boiled at 100°C for 10 min and rapidly cooled. The sample was mixed 1:1 with acid butanol (5% v/v) (Sigma) and free sialic acid was measured at OD 549/OD 532 nm.

### Analysis of Sialic Acids From Neuraminidase Treated Cells

HEK cells (5 × 10^6^ cells/ml) were incubated with 10% (v/v) *S. pneumoniae* culture supernatant for 30 min at 37°C in round bottom 96-Well polystyrene plates (Nunc, Roskilde, Denmark) in RPMI-HSA and centrifuged at 300 × g for 10 min at 4°C. Maackia Amurensis Lectin II (MAL-II) (Vector Labs, Burlingame, CA, US) was labeled with NT-647 dye following the instructions provided (NanoTemper, München, Germany). Washed cells were incubated with 45 nM NT-647 labeled MAL-II for 45 min at 4°C. Cells were washed by centrifugation and fixed with 1% (v/v) paraformaldehyde (Thermo Fisher Scientific, Waltham, MA, US) in RPMI-HSA. Events were acquired with BD LSR Fortessa flow cytometer (Laser 640 nm, filter 670/30) and analyzed using FlowJo 10.1r5 (FlowJo LLC, Ashland, Oregon). Gating of the cells was done with forward scatter (FSC) and side scatter (SSC) to find viable, single cell events.

For peripheral blood monocytic cell, erythrocyte, and platelet assays blood was drawn to hirudin (Roche Diagnostics, Mannheim, Germany) or citrate tubes from healthy human volunteers after informed written and signed consent (Ethical Committee decision 406/13/03/00/2015, Hospital district of Helsinki and Uusimaa). Blood was diluted 1:1 with PBS and then incubated 1:1 with *S. pneumoniae* culture supernatant in round bottom polystyrene tubes (Corning Inc., NY, US) in an orbital rotator for 1 h at 37°C. Next, cells and platelets were isolated by centrifugation through a gradient (Histopaque 1.119 and 1.077; Sigma-Aldrich) as previously described (Nissila et al., 2018). Cell, erythrocyte and platelet layers were collected in separate tubes, washed once with RPMI-HSA and diluted to RPMI-HSA. Cells (5 × 10^6^ cells/ml) or platelets were incubated with 45 nM of NT-647 labeled MAL-II lectin similarly as described above.

### Analysis of Galactose From Neuraminidase Treated Cells

Fluorescein-labeled lectin from *Arachis hypogaea* (Peanut agglutinin) (FITC-PNA) (Sigma-Aldrich, St. Louis, MI, US) was used to analyze exposed galactose on the neuraminidase treated cells. Cells were treated with *S. pneumoniae* recombinant neuraminidase (RecNanA) in RPMI-HSA. Cells were pelleted at 300 × g for 10 min at 4°C, the pellet was resuspended in RPMI-HSA medium and incubated with FITC-PNA. Events were recorded with BD LSR Fortessa (laser 525/50).

### Platelet Activation and Platelet Aggregation Assays

Blood was collected in citrate tubes (BD Vacutainer®, Franklin Lakes, NJ, US), diluted with PBS in 1:1 volume and incubated with *S. pneumoniae* supernatant (1:1 volume) and for 60 min at 37°C. After centrifugation at 200 × g, 20 min at room temperature (acceleration = 0, brake= 0). Platelet-rich plasma was collected in citrate buffer (9.4 mM citrate, 4.8 mM citric acid, 17.4 mM dextrose, 145 mM NaCl, pH 6.5) and washed twice at 440 × g (20 min, room temperature, acceleration = 0, brake = 0). After wash platelet-rich plasma was resuspended in modified Tyrode's buffer (137 mM NaCl, 2.7 mM KCl, 1 mM MgCl_2_, 7 mM HEPES, 0.35% BSA, 5.5 mM dextrose, 2 mM CaCl_2_, pH 7.4). Platelets were activated by adding hirudin plasma from the same donor (33% v/v) and incubated 15 min at 37°C. Activation was blocked by adding citrate buffer. Plasma was removed by centrifugation as before. Pellet was resuspended and stained with PeCy5 labeled antiCD62p (BioLegend, San Diego, CA, US) and FITC labeled CD41a (Invitrogen, Carlsbad, CA, US) for 10 min at room temperature. Events were recorded with BD LSR Fortessa (laser 670/30) and analyzed with FlowJo 10.1r5.

Aggregation assay was modified from a previously published protocol (Hyvarinen and Jokiranta, 2015). Platelet suspension was prepared in modified Tyrode's buffer (200 × 10^6^ particles/ml). Supernatants from *S. pneumoniae* strains were added to the platelets (10 % V/V) and incubated at room temperature for 10 min in 96-well black clear-bottom microplates (Perkin Elmer). Absorbance at 405 nm was recorded immediately in Hidex Sense Microplate Reader for single particles (double orbital shaking mode 120 rpm, 26°C, 30 s intervals, 10 min incubation). Hirudin plasma from the same individual was added 17% (V/V) and further absorbance 405 nm reading was made.

### Hemolysis Measurements and Complement Activation in Whole Blood

*S. pneumoniae* culture supernatants (1:1 volume) were incubated with hirudin anticoagulated blood as described above. After incubation the samples were centrifuged for at 300 × g for 20 min at 22°C to separate the plasma. Hemolysis was measured from the 1:1 PBS diluted plasma by observing release of hemoglobin at 405 nm. To analyze the complement activation levels separated plasma was supplemented with 10 mM of EDTA to stop further complement activation and stored at −20°C. Soluble C5b-9 concentrations were measured from the serially diluted plasma using MicroVue C5b-9Plus EIA kits (Quidel, San Diego, CA), according to the manufacturer's instructions.

### Factor H Removal Assay

To examine if recNanA could remove cell surface bound Factor H, 300 μl of the U937 cells at 5 × 10^6^ cells/ml concentration in RPMI-HSA were preincubated with 300 nM NT647-labeled factor H (Complement Technology) at 4°C for 45 min. After preincubation, cells were washed with 1.2 ml RPMI-HSA and centrifugated (300 × g, 10 min) and resuspended in 600 μl 1% (v/v) paraformaldehyde in RPMI-HSA and divided in half. Incubation of both samples was continued at 37°C either in the presence or absence of 150 nM recNanA. To detect cell-bound factor H during incubation, fluorescence intensities of the cells were measured with BD LSR Fortessa flow cytometer (650 nm) at different time points: 0, 2, 4, 8, 16, 32 (min). Acquired data was analyzed with FlowJo 10.1r5 as described above.

### The Effect of Recnana on Pneumolysin Mediated Hemolysis

Human blood collected in hirudin tubes was centrifugated at 650 × g for 10 min at 4°C to isolate the red blood cells. After removing plasma cells were washed twice with ~1.5 ml ice-cold PBS (2,000 rpm for 10 min at 4°C) and finally diluted 1:100 in PBS. Hemolysis was measured following incubation with different concentrations (0, 5, 10, 20, and 60 nM) of pneumolysin (PLY) from *S. pneumoniae* serotype two (strain D39 / NCTC 7466) that was purchased from Cusabio (CUSABIO TECHNOLOGY LLC, Houston, TX, US). A 2-fold (1:2) RecNanA dilution series was prepared from 800 to 0 nM. Cells were incubated with these different RecNanA concentrations and a specific PLY concentration (0, 5, 10, 20, and 60 nM) in a total volume of 50 μl for 30 min at 37°C. Hemolysis was measured by pelleting the cells (300 × g, 10 min), diluting 30 μl of the supernatant with 70 μl PBS and measuring their absorbance at 405 nm with Labsystems iEMS Reader MF.

### Statistical Measurements

Statistical significance between repeated experiments was analyzed using one-way ANOVA pair-wise comparison or one-way ANOVA with Tukey's *post hoc* test when multiple comparisons were made (SPSS version 24 IBM Statistics) by using a standard *p*-value threshold of <0.05 as indicating statistical significance. Standard deviations of repeated experiments are shown by error bars.

## Results

### *S. pneumoniae* RecNanA Removes Sialic Acids From Cells

**Figure 1:**
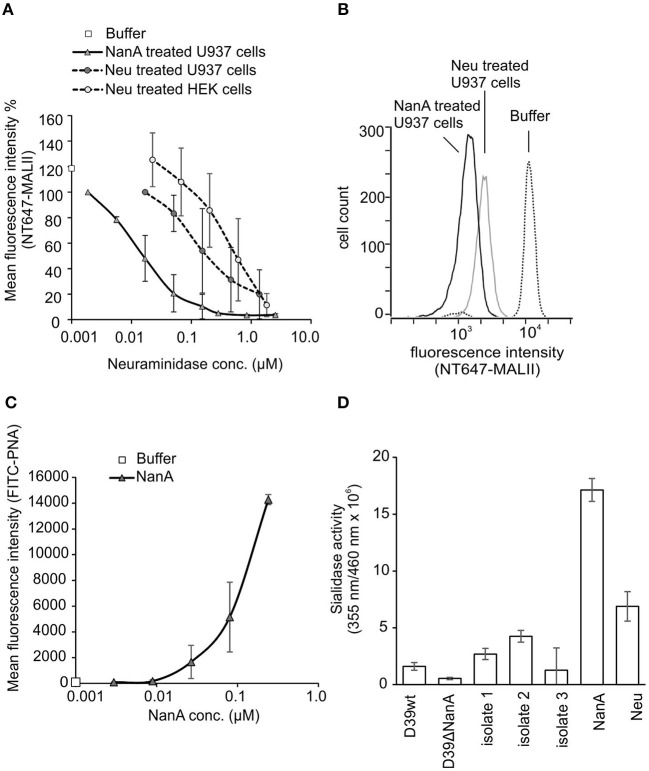
Removal of sialic acids from cell surfaces by RecNanA (NanA) and *C. perfringens* neuraminidase (Neu). 2.2 × 10^4^ of U937 or HEK cells were incubated with increasing concentrations (nM range) of NanA or Neu. Sialic acid removal was detected by flow cytometry using **(A,B)** sialic acid-specific NT647-labeled MAL-II lectin (*n* = 2) or **(C)** galactose-specific peanut agglutinin (PNA) (*n* = 2). **(A)** Levels were calculated as relative mean fluorescence intensity of NT647-MAL-II without neuraminidase. **(B)** Histogram showing distribution of MALII binding on U937 cells in the presence of 300 nM NanA, Neu or buffer. **(C)** Increase of FITC-PNA binding upon removal of sialic acids was detected with increasing concentrations of NanA. **(D)** Sialidase activities of the purified proteins RecNanA (10 nM of NanA) and *C. perfringens* neuraminidase (110 nM of Neu) and the bacterial supernatants (50%) from *S. pneumoniae* wild type D39 strain D39 *nanA* (D39ΔnanA) mutant and the clinical isolates. Error bars indicate SD.

To study sialic acid removal by RecNanA from cells, increasing concentrations of RecNanA were incubated with U937 cells. The presence of sialic acids was detected by using NT647-labeled MAL-II that recognizes α-2-3 linked terminal sialic acids. Binding of NT-647-MALII was completely inhibited after treatment of U937 cells with 30 nM of RecNanA, while 1.3 μM of *C. perfringens* neuraminidase (Neu) was required for the same effect (Figures 1A,B). To verify cleavage of sialic acids from the U937 cells the exposure of terminal galactose upon RecNanA incubation was detected using PNA (Figure 1C). Increasing concentrations of RecNanA resulted in a clear dose-dependent increase in PNA binding to cells indicating that sialic acids were released from the cells.

To determine the sialidase activities of the purified proteins and the bacterial supernatants used in this study we performed a fluorogenic neuraminidase assay using 2′-(4-Methylumbelliferyl) -α-D-N-acetylneuraminic acid (MUAN) as a substrate. The sialidase activities of RecNanA (NanA) and *C. perfringens* neuraminidase (Neu) were consistent with the NT-647-MALII assay showing at least 10-fold higher activity for RecNanA than Neu (Figure 1D).

### NanA in *S. pneumoniae* Culture Supernatant Cleaves Sialic Acids From Cell Surfaces

**Figure 2:**
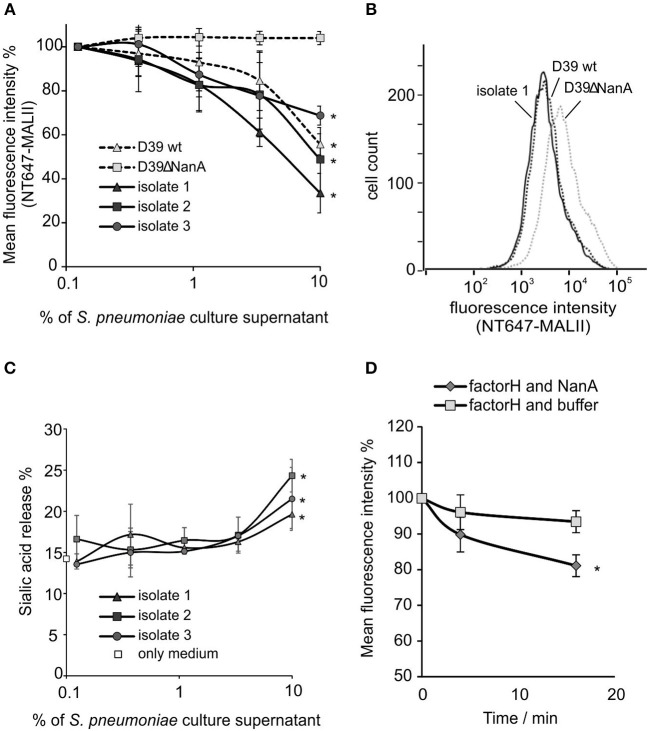
Removal of sialic acids from cell surfaces by *S. pneumoniae* culture supernatants. 5 × 10^6^ HEK cells were incubated with increasing volumes (% of supernatant in total volume of the sample) of *S. pneumoniae* late log-phase culture supernatants from wild type D39 strain (D39 wt), D39 *nanA* deletion mutant (D39ΔNanA) and three clinical isolates (isolate 1–3). Sialic acid removal was detected by flow cytometry using **(A,B)** sialic acid specific NT647-labeled MAL-II lectin or **(C)** by detecting free sialic-acid in the supernatant by thiobarbiturate acid at 450/532 nm. **(B)** Histogram showing distribution of MALII-binding on HEK cells in the presence of supernatants from D39 wt, D39ΔNanA and isolate 1. Levels are calculated as **(A)** relative mean fluorescence intensity of NT647-MAL-II without culture supernatant (*n* = 3) or **(C)** with 1.3 μM of *C. perfringens* neuraminidase. **(D)** Removal of factor H from U937 cell surface by NanA. Fluorescence intensities of U937 cells, that were preincubated with 300 nM NT647-labeled factor H, were detected with flow cytometry at different time points during incubation of the cells with or without NanA (150 nM) (*n* = 3). Relative mean fluorescence intensities of the samples are shown. Statistical significance **(A)** against MALII binding to D39ΔNanA cells or **(C)** sialic acid release % **(D)** or FH binding with only medium or buffer was calculated using One-way ANOVA (*P-value < 0.05). Error bars indicate SD.

To study whether released NanA is enzymatically active HEK cells were incubated with *S. pneumoniae* culture supernatants from two laboratory strains, a wild type D39 strain and its *nanA* mutant. Because all *S. pneumoniae* strains are known to express NanA, sialic acid removal by culture supernatants was studied also from three clinical *S. pneumoniae* isolates (King et al., 2005). The wild type D39 strain (D39 wt) and all three clinical isolates showed a significant reduction in NT647-MALII binding, when compared to the D39 *nanA* (D39ΔnanA) mutant indicating that NanA is active in the full bacterial secretome (Figures 2A,B). Moreover, incubation of the cells with the supernatants from three clinical isolates resulted in gradually elevated levels of free sialic acids when compared to the background sample with medium only (Figure 2C). Because FH is known to bind to sialic acids we next studied whether removal of sialic acids by NanA reduces binding of FH on the U937 cells. Here, a significant reduction of FH on cell surfaces could be detected after a 15 min incubation with NanA when compared to the buffer control (Figure 2D).

**Figure 3:**
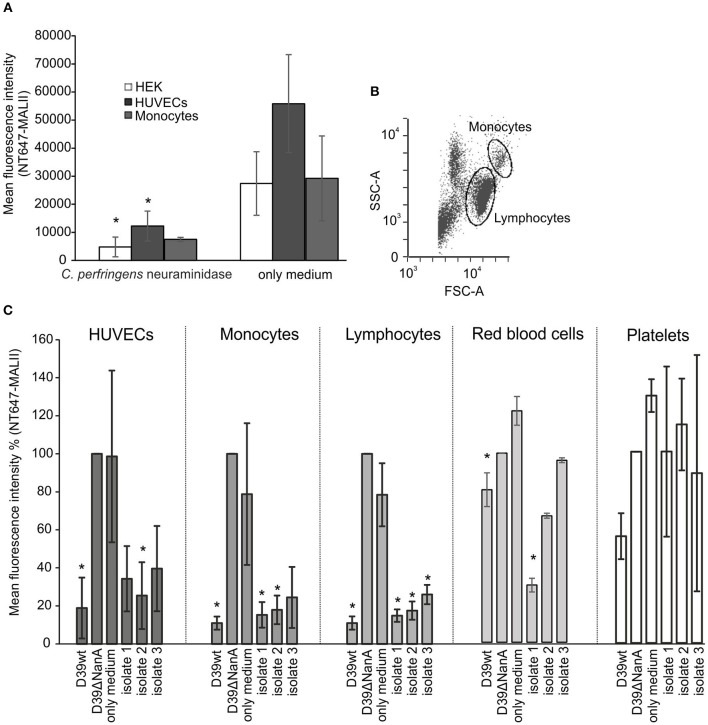
Removal of sialic-acid by *S. pneumoniae* NanA from various cells. Cultured HEK and HUVEC cells and hirudin-anticoagulated whole blood were incubated with **(A)** *C. perfringens* neuraminidase (*n* = 3) and **(C)** *S. pneumoniae* culture supernatants from wild type D39 strain (D39 wt), D39 *nanA* deletion mutant (D39ΔNanA) and three clinical isolates (isolates 1–3) (*n* = 3). Red blood cells, peripheral blood monocytic cells, and platelets were isolated from whole blood using double density gradient centrifugation. **(B)** Gating of the monocytes and lymphocytes from the peripheral blood monocytic cells are shown. **(A)** Sialic acid removal from HEK and HUVEC cells and monocytes was detected by flow cytometry using sialic acid specific NT647-labeled MAL-II lectin. Levels are calculated as **(A)** mean or **(C)** relative mean fluorescence intensity against NT647-MAL-II binding to D39ΔNanA treated cells. Statistical significance calculated for HEK, HUVEC, red blood cells, and peripheral blood monocytic cells (*n* = 3) **(A)** against MALII binding with only medium or **(B)** NT647-MAL-II binding to D39ΔNanA cells using one-way ANOVA (*P-value < 0.05). Error bars indicate SD.

Because of the known variation of sialic acid expression on different cell types (Nissila et al., 2018) we next studied the ability of neuraminidase and *S. pneumoniae* culture supernatants to cleave sialic acids from different cell lines (HUVEC, HEK-293T) and human cells (monocytes, lymphocytes, red blood cells) and platelets in peripheral blood. Incubation of HEK and HUVEC cells in growth medium with 2 μM of *C. perfringens* neuraminidase resulted in removal of sialic acids (Figure 3A) when compared to cells that were incubated in growth medium only. Also isolated red blood cells and monocytes showed a slight reduction in their surface sialic acid content after incubation with the enzyme (2 μM). The *S. pneumoniae* culture supernatant from D39 wild type strain removed surface sialic acids from HUVEC cell lines as well as from monocytes and lymphocytes in whole blood (Figure 3C), while no cleavage of sialic acids could be detected by the supernatant from D39 *nanA* mutant. Moreover, *S. pneumoniae* culture supernatants from the clinical isolates 1 and 2 removed sialic acids from monocytes and lymphocytes in whole blood while only isolate 2 showed significant removal of sialic acids from HUVEC cells and only isolate 1 showed significant removal of sialic acids from red blood cells. These results suggest that the release of NanA by *S. pneumoniae* is able to remove terminal sialic acids from various cell types in whole blood in the presence of whole bacterial secretome.

### *S. pneumoniae* NanA Increases Platelet Aggregation in Whole Blood

**Figure 4:**
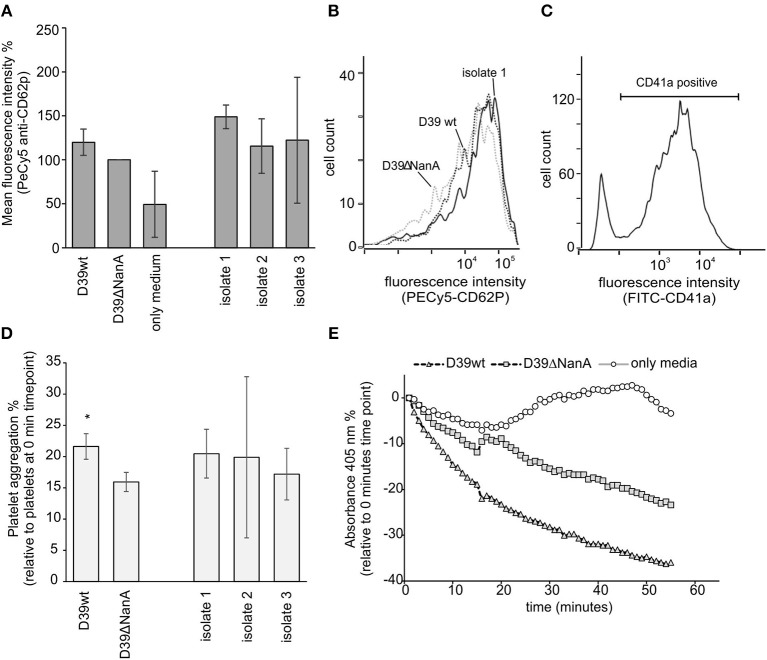
Effect of *S. pneumoniae* NanA expression on platelet activation and aggregation. **(A–C)** Bacterial culture supernatants from *S. pneumoniae* D39 wild type (D39 wt), D39 mutant and three clinical isolates (isolate 1–3) were incubated in citrate anticoagulated whole blood. **(A)** Activation of platelets was detected by analyzing the expression of CD62P (P-selectin) by flow cytometry (*n* = 3). Intensity levels are calculated as relative mean fluorescence intensity against CD62P expression by D39ΔNanA supernatant treated cells. **(B)** A histogram showing distribution of CD62P-binding on platelets in the presence of supernatants from D39 wt, D39ΔNanA and isolate 1. **(C)** Gating of platelets using anti-CD41a antibody. **(D)** To detect platelet aggregation platelets were isolated and the samples were treated with hirudin plasma after 10 min incubation with bacterial supernatant (*n* = 3). Aggregation of platelets was determined by subtracting the absorbance values at 0 time point from the absorbance values at the end time point shown in **(E)**. Statistical significances are calculated against **(A)** CD62P expression or **(D)** aggregation between D39ΔNanA and D39 wild type supernatant treated cells using one-way ANOVA (*P-value <0.05). Error bars indicate SD.

Disturbed recognition of sialic acids by factor H on endothelial cells and platelets is considered to be the key mechanism causing direct complement attack against these cells and contribute to thrombotic microangiopathy in aHUS (Hyvarinen et al., 2016). Therefore, we next determined the role of sialic acid removal by *S. pneumoniae* NanA in the activation and aggregation of platelets in whole blood. When sodium citrate anticoagulated whole blood was incubated with *S. pneumoniae* culture supernatants from D39 wt, D39 *nanA* mutant (D39 ΔNanA) and three clinical isolates no significant increase in CD62P expression was observed between the strains (Figures 4A–C). However, we observed a significant increase in platelet aggregation by culture supernatants from D39 wt when compared to the aggregation by supernatant from D39 ΔNanA strain (Figures 4D,E) indicating that desilaylation by NanA has a role in platelet aggregation.

### *S. pneumoniae* NanA Increases Complement and Pneumolysin-Mediated Hemolysis in Whole Blood

**Figure 5:**
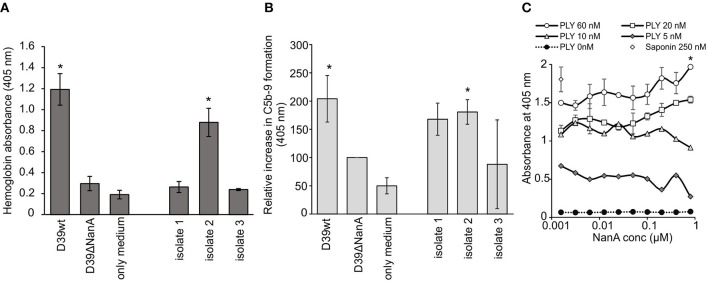
Effect of *S. pneumoniae* NanA expression on complement-mediated red blood cell lysis. **(A)** Hirudin-anticoagulated whole blood was incubated for 1 h at 37°C with the supernatants from *S. pneumoniae* D39 wild type (D39 wt), D39 *nanA* deletion mutant (D39ΔNanA) and three clinical isolates (isolates 1–3). Hemolysis was determined by measuring absorbance of hemoglobin at 405 nm from the plasma (*n* = 3). **(B)** Complement activation in whole blood was determined by measuring the formation of soluble SC5b-9 from blood isolated plasma by EIA (450 nm) (*n* = 3). All values are shown as a percentage of A450 values relative to D39 *nanA* mutant strain. Increase in SC5b-9 is calculated as relative absorbance against D39ΔNanA supernatant treated blood that was 0.500 ± 0.150 at 450 nm in the isolated plasma. **(C)** Effect of NanA on hemolysis caused by pneumolysin (PLY). Red blood cells were incubated with increasing concentration of NanA compared with specific PLY concentration (0, 5, 10, 20, or 60 nM). Hemolysis was detected by measuring absorbance of the supernatant at 405 nm. Absorbances for different PLY concentrations (0–60 nM) are shown (*n* = 4). Saponin was used as a positive control and sample without PLY as negative control (0 nM PLY) for red blood cell hemolysis. Statistical significances are calculated against **(A,B)** D39ΔNanA supernatant treated blood **(C)** or between the highes and lowest NanA concentration using one-way ANOVA (*P-value <0.05). Error bars indicate SD.

A common clinical presentation in aHUS is intravascular hemolysis that is induced by complement attack against autologous cells. Therefore, we next determined the role of sialic acid removal by NanA in red blood cell lysis and complement activation *ex vivo* in whole blood. Incubation of hirudin-anticoagulated blood with supernatants of *S. pneumoniae* D39 wild type (D39 wt) and one clinical *S. pneumoniae* isolate resulted in a significant increase in plasma hemoglobin (Figure 5A), while no increase could be detected with the supernatant of the D39 ΔNanA. Similarly, incubation of supernatant of D39 wt in whole blood showed a significant increase in plasma SC5b-9 when compared to the sample incubated with supernatant of the D39 ΔNanA (Figure 5B). In addition, incubation of supernatant of *S. pneumoniae* clinical isolate 2 resulted in a significant increase in plasma SC5b-9. These results indicate that the expression of NanA by *S. pneumoniae* increases complement-mediated hemolysis. Although, we could detect significant differences between the hemolytic activities of *S. pneumoniae* D39 wild type (D39 wt) and D39 ΔNanA supernatants, we could not rule out the possibility that NanA could have an effect on pneumolysin mediated hemolytic activity as well. Therefore, we next performed a hemolysis assay in the presence of pneumolysin and increasing concentrations of NanA. A dose dependent increase in pneumolysin mediated hemolysis could be detected in the presence of increasing concentrations of NanA while NanA itself did not cause any hemolysis (Figure 5C). However, in limited pneumolysin concentrations (below 20 nM) no dependence on NanA concentrations on pneumolysin mediated hemolysis could be detected.

## Discussion

Pneumococcal HUS is one of the most severe complications of invasive pneumococcal disease (Janapatla et al., 2013). Pneumococcal neuraminidase activity has been suggested to trigger pHUS (Coats et al., 2011) but it is unclear whether expression of neuraminidase correlate with HUS disease pathology (Janapatla et al., 2013; Smith et al., 2013; Singh et al., 2016). In this study, we demonstrate that the presence of NanA increases the ability of *S. pneumoniae* to cleave sialic acids from host cells in the presence of whole blood and whole bacterial secretome. Removal of host sialic acids from host cells by the enzyme resulted in complement activation in blood plasma, enhanced platelet aggregation and hemolysis *ex vivo*.

Here, 30 nM of *S. pneumoniae* RecNanA was required to remove nearly 100% of sialics from cell surfaces (Figure 1). The *C. perfringens* neuraminidase is known to reduce sialic acid recognition by factor H on self cells (Nissila et al., 2018) indicating that NanA can potentially disturb this interaction at low expression levels and thereby trigger AP activation against self cells and lead to thrombosis and hemolysis in a similar mechanism as in aHUS (Hyvarinen et al., 2016).

NanA has the potential of cleaving sialic acids from various cell types (Klein et al., 1977; Parker et al., 2009; Smith et al., 2013) and, as shown here, also in the presence of whole blood and the whole bacterial secretome (Figures 2, 3). It is known that host proteins contain N-glycan structures and therefore removal of sialic acids by NanA could also alter the function of these proteins in our *ex vivo* assay (King et al., 2004). However, this would probably not affect the function of factor H, as N-linked glycans in factor H have structural rather than functional roles in the interactions between factor H and its natural binding partners (Fenaille et al., 2007).

In HUS pathogenesis, platelet activation, endothelial cell damage, and hemolysis are considered hallmarks of the disease. In pHUS the morbidity and mortality is higher than in classical STEC-HUS having also a poorer long-term prognosis (Smith et al., 2013). This indicates that microbial virulence factors contribute to the severity of the disease. In our hands the presence of NanA produced by the D39 strain efficiently removed sialic acids and led to complement activation, platelet aggregation and hemolysis in whole blood when compared to the NanA deletion mutant. Although this was not that evident with the studied clinical isolate supernatants it has been previously shown that exposure of platelets to soluble NanA leads to platelet hyper reactivity (Kullaya et al., 2018).

All clinical isolates tested express NanA, but according to previous studies the expression or activity of this enzyme does not correlate with the disease state (Singh et al., 2016). All the supernatants from clinical isolates were able to remove sialic acids from cells. Incubation of whole blood with the supernatants from clinical isolates 1 and 2 resulted in a significant increase in complement activation that was detected as an increase in soluble C5b-9 in the isolated plasma. According to our results it is evident that the presence of NanA is crucial for the pathogenetic effects in our *ex vivo* model as it allows the carrier strain to remove sialic acids, activate complement and platelets, and cause hemolysis.

A potent cytolysin of *S. pneumoniae* is pneumolysin (Bokori-Brown et al., 2016). It is a cholesterol dependent pore-forming toxin known to be expressed by all *S. pneumoniae* strains (Kanclerski and Mollby, 1987). Therefore, it is possible that hemolysis observed in our assays could have partially been caused by this toxin. In this study we observed that NanA could alter the lytic activity of the toxin on self cells. The reason for this could be that the recognition of cells by pneumolysin is partially dependent on specific cell exposed glycan structures (Shewell et al., 2014). The supernatant from the *nanA* mutant strain did not, however, lead to hemolysis in whole blood indicating that the hemolysis was at least partially caused by complement directed against desialylated self cells especially because removal of sialic acids reduced binding of factor H on self cells. Surprisingly, incubation of the supernatant from isolate 1 did not cause hemolysis in whole blood although it showed high sialidase activity on the studied cells. Therefore, we cannot rule out the possibility that increased susceptibility to complement-mediated hemolysis could have been caused by other factors than the neuraminidase enzyme. Previous studies have shown that Thomsen-Friedenreich antigen is exposed upon treatment with neuraminidase, particularly with NanA (Coats et al., 2011). Exposure of this antigen can lead to agglutination of erythrocytes and may thereby accelerate hemolysis (McGraw et al., 1989) and clearance of erythrocytes and platelets (Crookston et al., 2000; Coats et al., 2011; Janapatla et al., 2013).

Based on our results it is likely that NanA is crucial for sialidase activity against cells as the supernantant from *nanA* deletion mutant showed no increase in plasma C5b-9 that was isolated from supernatant incubated whole blood. This could be due to the broad sialic acid specificity of NanA that can cleave terminal sialic acids with α2–3, α2–6, and α2–8 linkages (Xu et al., 2011). Altough, NanB cleaves α2–3, α2–6, and α2–8, it has more activity on α2–3 while NanC is specific only for α2–3 linkages (Xu et al., 2008; Owen et al., 2015). Interestingly, the only sialic acid binding site in factor H domain 20 interacts with α2-3-sialylated glycans but the protein itself is α2-3- and α2-6-sialylated in a ratio of 8.5 and 91.5%, respectively (Blaum et al., 2015; Schmidt et al., 2018). Thereby, NanA could also act on factor H sialic acids and, speculatively, reduce stability of factor H.

**Figure 6:**
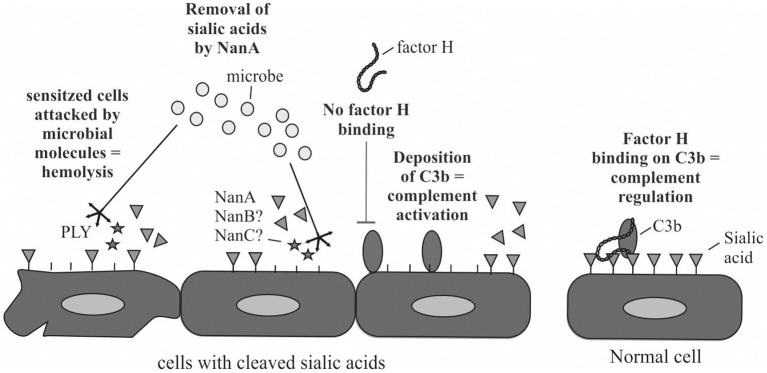
Schematic illustration of the putative mechanism how NanA could reduce complement regulation by factor H on the cells and sensitize cells for attack by complement and thereby lead to cell damage. In addition, NanA could increase the susceptibility of cells to attack by pneumolysin (PLY) or other virulence factors.

Increase in complement activation in whole blood by the bacterial supernatant was detected from plasma that was isolated from whole blood after incubation with the supernatants. Soluble C5b-9, that is a byproduct of complement activation, was used as a marker for complement activation. Because of the significant correlation between NanA expression and increase in blood isolated plasma C5b-9 these results suggest that NanA increases complement activity in whole blood by cleaving sialic acids from cell surfaces and predisposes sensitized red blood cells for complement and pneumolysin mediated lysis and platelets for aggregation (Figure 6). Thereby, NanA could contribute to pHUS pathogenesis. This effect could be especially strong in individuals with predisposing genetic variants in complement genes such as *CFH* and *CFI* (Szilagyi et al., 2013).

## Data Availability

The datasets generated for this study are available on request to the corresponding author.

## Ethics Statement

Isolates 1, 2, and 3 were *S. pneumoniae* serotype two strains isolated from a blood culture of septic patients with the permission of the ethical review board of the Hospital District of Helsinki and Uusimaa, Finland (448/13/03/00/09).

For peripheral blood monocytic cell, erythrocyte and platelet assays blood was drawn to hirudin (Roche Diagnostics, Mannheim, Germany) or citrate tubes from healthy human volunteers after informed written and signed consent (Ethical Committee decision 406/13/03/00/2015, Hospital district of Helsinki and Uusimaa).

## Author Contributions

SS helped in data interpretation and manuscript evaluation, designed the analysis, wrote the paper, and performed analysis. PH and HL performed analysis. AS contributed data or analysis tools. SK, SM, and TJ helped in data interpretation, helped to evaluate and edit the manuscript, contributed data or analysis tools. KH supervised development of work, helped in data interpretation and manuscript evaluation, designed the analysis, wrote the paper, and performed analysis.

### Conflict of Interest Statement

KH was employed by company ThermoFisher Scientific, Vantaa, Finland and TJ by company SYNLAB Helsinki, Finland. The remaining authors declare that the research was conducted in the absence of any commercial or financial relationships that could be construed as a potential conflict of interest.
